# Physical–Mechanical Characteristics and Microstructure of Ti6Al7Nb Lattice Structures Manufactured by Selective Laser Melting

**DOI:** 10.3390/ma13184123

**Published:** 2020-09-16

**Authors:** Cosmin Cosma, Igor Drstvensek, Petru Berce, Simon Prunean, Stanisław Legutko, Catalin Popa, Nicolae Balc

**Affiliations:** 1 Department of Manufacturing Engineering, Technical University of Cluj-Napoca, 400641 Cluj-Napoca, Romania; petru.berce@tcm.utcluj.ro (P.B.); nicolae.balc@tcm.utcluj.ro (N.B.); 2 Faculty of Mechanical Engineering, University of Maribor, 2000 Maribor, Slovenia; igor.drstvensek@um.si; 3 Faculty of Sciences, University of Southern Denmark, 5000 Odense, Denmark; simon.sdu.dk@gmail.com; 4 Faculty of Mechanical Engineering, Poznan University of Technology, 60-965 Poznan, Poland; stanislaw.legutko@put.poznan.pl; 5 Department of Materials Science and Engineering, Technical University of Cluj-Napoca, 400641 Cluj-Napoca, Romania; catalin.popa@stm.utcluj.ro

**Keywords:** lattice structure, porosity, compressive strength, Young modulus, mathematical prediction, microscopic analysis

## Abstract

The demand of lattice structures for medical applications is increasing due to their ability to accelerate the osseointegration process, to reduce the implant weight and the stiffness. Selective laser melting (SLM) process offers the possibility to manufacture directly complex lattice applications, but there are a few studies that have focused on biocompatible Ti6Al7Nb alloy. The purpose of this work was to investigate the physical–mechanical properties and the microstructure of three dissimilar lattice structures that were SLM-manufactured by using Ti6Al7Nb powder. In particular, the strut morphology, the fracture characterization, the metallographic structure, and the X-ray phase identification were analyzed. Additionally, the Gibson-Ashby prediction model was adapted for each lattice topology, indicating the theoretical compressive strength and Young modulus. The resulted porosity of these lattice structures was approximately 56%, and the pore size ranged from 0.40 to 0.91 mm. Under quasi-static compression test, three failure modes were recorded. Compared to fully solid specimens, the actual lattice structures reduce the elastic modulus from 104 to 6–28 GPa. The struts surfaces were covered by a large amount of partial melted grains. Some solidification defects were recorded in struts structure. The fractographs revealed a brittle rupture of struts, and their microstructure was mainly α’ martensite with columnar grains. The results demonstrate the suitability of manufacturing lattice structures made of Ti6Al7Nb powder having unique physical–mechanical properties which could meet the medical requirements.

## 1. Introduction

The evolution of additive manufacturing (AM) technology has led to the possibility of processing various macro-porous networks, so-called lattice structures. By integrating cellular gradients into medical applications, they can provide promising perspectives on the physical–mechanical behavior of implants [1]. Compared to fully solid implants, lattice applications can provide physical–mechanical characteristics tailored to the patient’s needs, adapting their macro-porosity and elasticity. Moreover, the lattice medical applications can make the AM production more efficient because the material consumption and the manufacturing time is reduced. By using conventional technologies such as investment casting, powder metallurgy, and metal foaming method, it is difficult to fabricate anatomical implants having integrated lattice structures that are geometrically defined [2,3,4,5]. The selective laser melting (SLM) process is part of AM and it is also known as laser powder bed fusion. This AM process is capable of directly producing complex fully dense components [6,7] and lattice structures made of metal alloys [8,9,10]. In the literature, typical lattice structures have been SLM-manufactured, and their mechanical properties have been investigated. Typical lattice structures tested were body-centered cubic, diamond, gyroid, and other triply periodical minimal surfaces [11,12,13,14,15]. Compared with conventional implants, the porous structures can stimulate the osseointegration process, limiting the rejection risk, and they can also facilitate the biological fluids to ingress [16,17]. On the other hand, a conventional implant may cause the stress shield after surgery and eventually result in the failure of it [18]. The stress shield appears because there is a stiffness mismatch between the conventional implant and human bone. For example, the elasticity modulus of conventional Ti6Al4V implants is about 110 GPa, which is significantly higher than that of human bone (10–29 GPa for cortical bone, and 0.8–5 GPa for trabecular bone). Previous studies have recommended the use of lattice structures to avoid the stress shielding effect. To improve the implant stability, the tissue bone can ingrowth creating a mechanical interlock into these porous structures [19,20]. In the last years, lattice bio-metallic implants were developed, using SLM technology, to treat total knee arthroplasty [21], chondrosarcoma localized in calcaneum [22], and maxillofacial reconstructions [23,24]. The results of these pilot surgical interventions are acceptable, with the lattice implants being integrated into the body without any signs of rejection and improving patients’ health.

From a material point of view, previous SLM research has predominantly focused on lattice specimens made of Ti6Al4V [25,26,27,28,29], CoCr [30,31], and stainless-steel alloy [10,32,33,34]. Thanks to the modified chemical composition, Ti6Al7Nb alloy is characterized by higher corrosion resistance and bio-tolerance in comparison with Ti6Al4V [35,36]. The Ti6Al7Nb alloy is gaining attention mainly thanks to weak solubility of niobium oxide in tissue environment and non-toxicity of oxide layers or soluble ions [37]. A review of published reports revealed that just one research study analyzed some Ti6Al7Nb lattice structures [37]. This study was limited to a single lattice topology (cubic), without providing the elasticity behavior. Consequently, new research on the mechanical response of Ti6Al7Nb lattice structures is demanded, being focused both on compressive strength and elasticity modulus. These mechanical characteristics are important to meet the medical requirements.

Moreover, using the Ti6Al7Nb powder could lead to multiple issues, and proper process parameters are needed for a stable fabrication of lattice structures. The information regarding the process parameters for this powder is also limited.

The purpose of this study was to investigate the physical–mechanical properties and the microstructure of three dissimilar lattice structures made of Ti6Al7Nb powder SLM processed. While maintaining the same porosity level, the considered designs of lattice structures were a commonly body-centered cubic structure and two new concepts. The lattice specimens were mechanical tested under static compression, and they were morphologically, metallographically, and structural analyzed. Additionally, the Gibson–Ashby mathematical model, which can estimate the Young modulus, and compressive strength was customized for each lattice topology. In order not to influence the mechanical results, the specimens were not subjected to any stress-relief treatment. Finally, a future approach is presented, describing a hybrid lattice implant which could satisfy the mechanical requirements of human bones.

## 2. Experimental Section

### 2.1. Powder

Due to the presence of vanadium in Ti6Al4V alloy, which can cause side effects around it after implantation, this study focused on Ti6Al7Nb, with a better expected biocompatibility [35,36,37]. The Ti6Al7Nb powder was produced by gas atomization, and, according to material data sheet, it has the following chemical composition: 84.9% Ti, 6.5% Al, 7.5% Nb, and other chemical elements under 0.5%. The particles’ diameter is between 20 and 63 µm (TLS Technik Spezialpulver, Bitterfeld-Wolfen, Germany). To improve the flowability of powder, the Ti6Al7Nb grains were treated at 500 °C in air for 1 h. The powder flow rate (s/50g) was measured by using a hall flowmeter funnel with an orifice of 2.5 mm. The hall flow rate was expressed as the time required for a 50 g powder specimen to be discharged by gravitational force through the flowmeter funnel [38]. After the heat treatment, the flow rate increased significantly from 68 s/50g to 23 s/50g. The X-ray (Inel, Artenay, France) spectroscopy demonstrated that the heat treatment did not alter the powder’s particle structure, but only the oxide state on the surface of particles [39].

### 2.2. Cell Design

**Figure 1:**
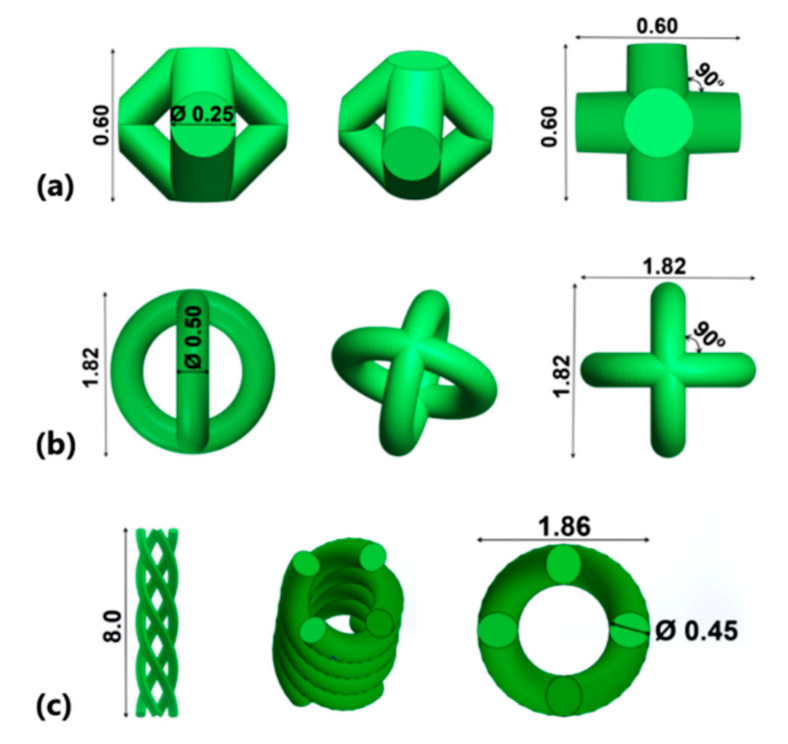
Virtual models of lattice topologies (mm): (**a**) body-centered cubic (BCC), (**b**) perpendicular circle intersection (PCI), and (**c**) parallel helix spirals (PHS).

In general, the lattice structures are characterized by periodic arrangements of open cells into a 3D model, providing distinct physical–mechanical properties. From a lattice-design point of view, the present topologies were generated by using the manual method. Manual generation means designing a lattice material by implementing beams and trusses with joints modified to create seamless transitions between unit cell elements [40]. Typical examples are simple cubic, body-centered cubic (BCC), face-centered cubic, diamond, octahedron, and rhombic dodecahedron [11,12,13,14,15,40]. This study takes into account three different topologies of lattice structures developed in Creo Parametric (Version 5.0, PTC, Boston, MA, USA) software, where each lattice design should contain 4 strut paths. A previous design-optimization study recommended that the strut diameter should range from 0.2 to 1 mm [18]. Besides, it was initially assumed that all the topologies should generate a macro-porosity about 60% in the lattice specimens. To archive this porosity level, a preliminary manufacturing investigation was conducted on each lattice topology. The first lattice unit considered was a common BCC structure with a reduced strut diameter. Setting the laser spot size at 45 µm and the process parameters detailed in Table 1, the reduced strut diameter obtained was 0.25 mm. To have the required porosity, the design space of the octahedral units is a cube with an edge length of 0.60 mm. Based on these findings, we design the present BCC structure showed in Figure 1a.

The new lattice concepts detailed above were elaborated in a such manner that during the SLM process, no support structures should be needed to sustain their struts (Figure 1b,c). When comparing with BCC structure, to increase the pore size of these new lattice units, their strut diameter should be higher. The second lattice unit contains a perpendicular circle intersection (PCI) and an isotropic design. The outer boundary diameter of the circle was fixed at 1.82 mm, and the strut diameter varied from 0.30 to 0.60 mm. The experimental fabrication test revealed that a strut diameter of 0.50 mm can generate the desired porosity. The features of PCI unit are detailed in Figure 1b. The third lattice unit has a bio-mimetic inspiration, like human deoxyribonucleic acid (or DNA), and has an anisotropic design (Figure 1c). It contains 4 parallel helix spirals (PHS) which make a complete 360° revolution in 8 mm height with a constant pitch. The outer boundary diameter was fixed at 1.86 mm, being similar to PCI unit. The strut diameter also varied between 0.30 and 0.60 mm, and the preliminary manufacturing evaluation shows that a strut diameter of about 0.45 mm is suitable.

**Figure 2:**
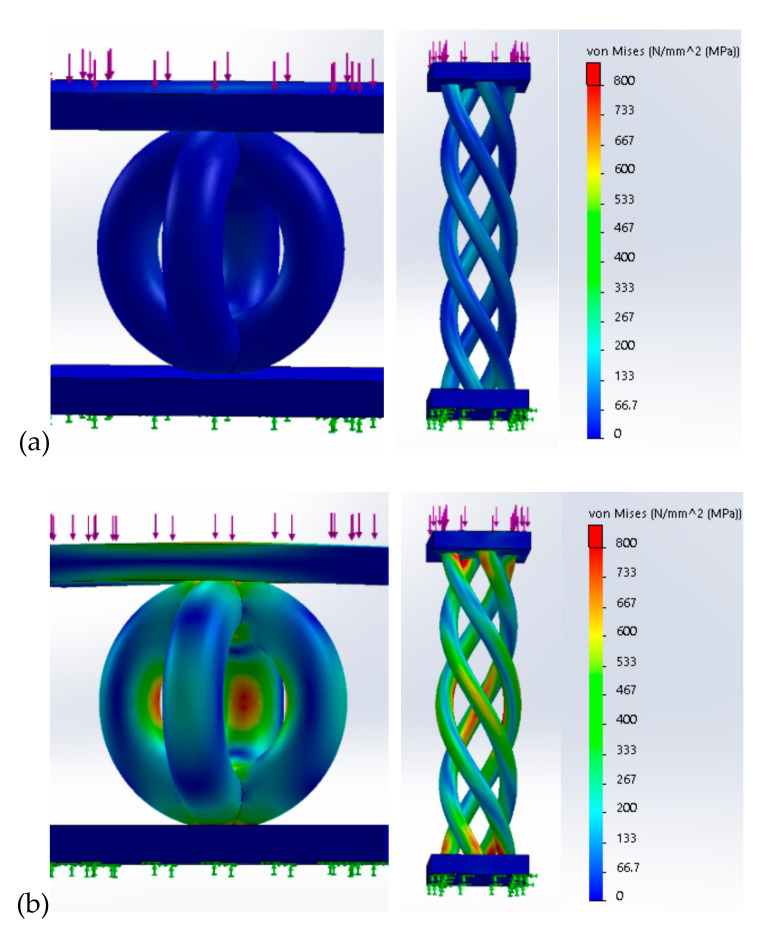
Von Mises stress distribution in PCI (right side) and PHS (left side) structure after applying a compressive load: (**a**) 10 N, (**b**) maxim force applied to reach the ultimate compressive strength of Ti6Al7Nb (approx. 790 MPa); 100 N for PCI structure, and 35 N for PHS structure.

Besides this preliminary manufacturing evaluation focused on porosity level, a finite element analysis (FEA) was elaborated. The FEA simulation can analytically predict the mechanical behavior of these lattice topologies. Because the numerical simulations are well documented for BCC structures [14,27], the FEA simulations were conducted just on the PCI and PHS units. The physical–mechanical properties applied are detailed in Section 3.2. A fine mesh was created on virtual models, and they were fixed on bottom surface (green arrows—Figure 2). Under compressive loads, the von Mises stress distribution is presented in Figure 2. Initially a compressive load of 20 N was applied on top surface (violet arrows—Figure 2a). The second simulation was completed, to establish the maxim load supported by these structures. It was theoretically determined by FEA that the PCI structure can support loads up to 100 N (Figure 2b). On the PHS structure, a limited load up to 35 N can be applied. At this uniaxial force, the PHS struts have a tension distribution that reached the ultimate compressive strength of Ti6Al7Nb SLM-manufactured (790 MPa). Under this theoretical load, the PHS lattice structures may fail. The FEA simulation demonstrates that the PCI structures could resist a superior compressive strength 3 times higher than the PHS specimens.

### 2.3. SLM Manufacturing

To obtain the cylinder specimens for mechanical trials, each lattice unit was multiplied several times, intersecting between them with 0.10 mm. The cylinder specimens had the following overall dimensions: the diameter of 12 mm and the height of 24 mm. The lattice cylinders were manufactured by a SLM 250 system (Realizer GmbH, Borchen, Germany). This equipment is capable of manufacturing medical grafts or implants directly from biometal powders, under the “slice by slice” principle. In this research, this technology can fuse the grains, using a solid laser up to 200 W power (type Nd:YAG). All the specimens were built randomly, being orientated vertical on the machine platform. The scanning strategy adopted was “X/Y”, which allows a scanning in X direction of “n” layer and in Y direction of “n + 1” layer. To anchor the bottom of lattice specimens, some support structures were prepared in RDesigner software (Version 1.1, Realizer GmbH, Borchen, Germany). The laser parameters programmed to print the support structures were 120 W laser power and 500 mm/s scanning speed. A soft recoater made of dense carbon fibers with Ø 7.5 µm was used to spread the powder. Because the SLM process involves heat transfer, remelting, and other thermo-physical behaviors, typical defects could occur in the Ti6Al7Nb lattice structures. Some of the typical manufacturing defects refer to the balling effect, micro-cracks, heat-affected zone, and residual stress phenomena [1,41,42,43]. To limit these issues, adapted process parameters are needed for a stable fabrication of lattice structures made of Ti6Al7Nb. After examining the literature recommendations regarding the SLM process parameters which could be proper for Ti6Al7Nb powder [20,35], and based on our previous studies [42,44,45,46], we programmed and tested more than 10 laser parameters. To have an efficient production speed, the layer thickness was maintained constant at 50 µm. In Table 1 are detailed the established process parameters which led to a good and stable manufacturing without evident defects in the specimens. Moreover, we calculated the total energy input per volume as a function of processing parameters [9]. These laser parameters were configured both for border and hatch scanning. Under a high-purity Ar-atmosphere and limited oxygen level (below 0.5%), the lattice specimens were printed. Ten specimens were produced for each lattice topology. To compare the mechanical properties, fully dense specimens were also fabricated with the same SLM conditions (Table 1). All the investigations were developed in as-built state, without conducting a stress-relief treatment or other heat treatments on specimens.

After the SLM fabrication, the unmelted powder from lattice pores was removed by ultrasounds, in an alcohol bath, at 40 °C for 1 h. The porosity of lattice cylinders was calculated by using Equation (1) [32]:

(1) $$P=\left(1\right.-\frac{w}{\pi \times r^{2}\times h\times \rho_{0}})\times 100\%\;\;\;\left(1\right)$$

where *P* is the porosity, *w* a weight, *r* and *h* are dimensions of the cylinder (radius and height), and *ρ*_0_ is the density of bulk Ti6Al7Nb. The present density of bulk Ti6Al7Nb was 4.50 g/cm^3^, being calculated on fully dense specimens manufactured with laser parameters detailed in Table 1. The weight of specimens was measured with an analytical balance (Partner AS 160.R2, precision ±0.2 mg).

### 2.4. Mechanical Testing and Estimation Equations

The mechanical tests were performed on fully dense and lattice specimens, using an Instron 1255 system (Instron, Norwood, MA, USA). This system has 500 kN maximum capacity, and the mechanical trials were completed at room temperature [47]. The specimens were loaded in the build direction, and they were tested with a 1 mm/min displacement rate, until mechanical failure occurred. During the uniaxial compression test, the displacement and reaction force were recorded to determine the compressive strength, strain, and Young modulus. The Young modulus characteristic is also known as elasticity modulus or stiffness. As previously explained, the porosity (or experimental density) directly influences the physical–mechanical characteristics, and this behavior is predominant in cellular structures [9]. The Gibson–Ashby mathematical model could estimate the longitudinal elastic modulus and the compressive strength of lattice structures as follows:

(2) $$E=E_{o}\times m\times P^{n}\;(\mathrm{GPa})$$

(3) $$\sigma =\sigma_{o}\times m\times P^{n}\;(\mathrm{MPa})$$

where *E*, *σ*, and *P* are the Young modulus, compressive strength, and porosity of lattice structures. *E*_0_ and *σ_o_* are corresponding to fully dense samples and represent the elasticity and the compressive strength. For a wide range of alloys, *n* exponent has been shown to vary from 1.8 to 2.3 and often is assumed to be 2 [10,21,26]. Additionally, the Equations (2) and (3) were adapted to each lattice topology, establishing experimentally the *m* coefficient, based on actual mechanical results. Thus, the *m* coefficient characterizes the dependence of elastic modulus and compressive strength on the porosity.

### 2.5. Microstructure and Metallographic Analyses

The morphological aspects of lattice specimens were investigated by scanning electron microscopy (SEM). The microscope used was Quanta 200 3D (FEI Technologies Inc, Hillsboro, OR, USA), and the accelerating voltage set up was 30 kV. During the SEM investigations, the Everhart–Thornley detector (ETD) was used in a high vacuum mode. Metallographic analyses were conducted, using an Olympus GX51 (Olympus Corporation, Tokyo, Japan) microscope, on ground and polished specimens, subsequently etched by Kroll’s reagent, for 6–10 s, at room temperature. Metallographic analysis of specimens was performed onto two directions, perpendicular and parallel to the layers (longitudinal and cross sections). The resulted lattice structures were studied by using an X-ray diffraction (XRD) system type Equinox 3000 (Inel, Artenay, France). The XRD analysis was carried out via Cu Kα radiation (λ = 1.54 Å).

## 3. Results and Discussion

### 3.1. Strut Diameter and Porosity Level

Geometric accuracy is often a limitation in fabrication of cellular structures, as is the entrapment of powder in the small pores’ spaces of these structures. In general, the geometric accuracy of SLM parts ranged from ±0.05 to ±0.10 mm. Thus, it is necessary to measure the strut size because the geometrical deviations are influenced by the process parameters. The cross-sectional metallographic investigations allowed us to measure the struts dimensions. Their mean value and standard deviation are summarized in Table 2. Similar dimensional deviations of struts were also reported [10,26,31,37]. Compared to the designed strut diameter, we see the printed one has increased values. The actual diameters of struts have increased values because they have many half-attached particles on their surfaces (details in Section 3.4). This fact influences the mechanical response of lattice specimens, but the impact is limited. Regarding the pore size, they were from 0.40 to 0.51 mm for BCC, from 0.50 to 0.62 mm for PHS, and from 0.85 to 0.91 mm for PCI specimens. The values were measured via SEM investigation.

The porosity of lattice models was calculated theoretically by Creo Parametric program, and it was 60% for all of them. After SLM manufacturing, the lattice specimens have a real porosity approximately 56%. The porosity of specimens is reduced because of the actual strut diameter, which was higher than the designed one. Compared to fully dense specimens, the lattice structures can reduce the processing time and the amount of powder required, making the SLM manufacturing more efficient. Furthermore, the osseointegration of macro-porous implants is highly influenced by the porosity level [48]. In vivo studies suggest that a macro-porosity over 50% can support the metabolic pathways and substrate transport mechanisms for a continuous bone tissue development inside of pores [49,50]. The present level of porosity matches this medical requirement.

### 3.2. Compression Properties

**Figure 3:**
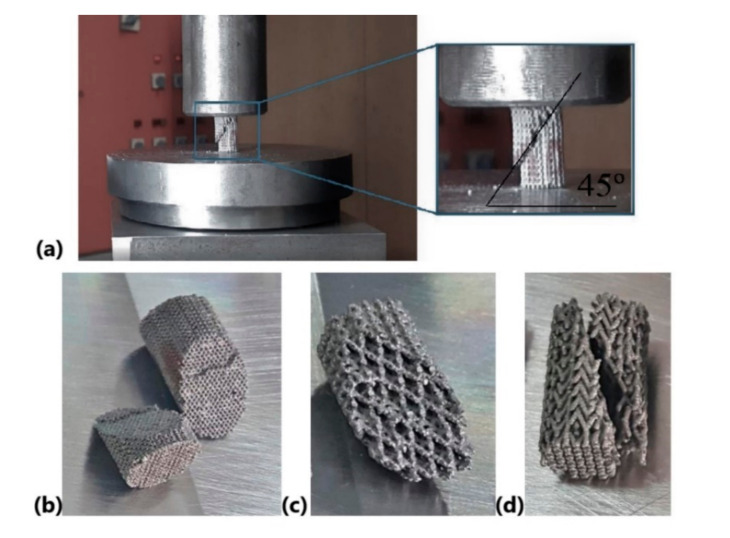
(**a**) Quasi-static compression test on Ti6Al7Nb specimens, (**b**) BCC, (**c**) PCI, and (**d**) PHS.

Before testing the lattice specimens, the compression properties of bulk Ti6Al7Nb were determined (Table 3). The fully dense specimens failed at approximately 790 MPa compressive stress and 104 GPa Young modulus. These fully dense specimens have a brittle behavior and a limited plastic deformation up to 1.5%. The brittle behavior of bulk Ti6Al7Nb specimens was also reported [35]. Figure 3a shows a lattice specimen during the quasi-static compression test, indicating the location and direction of fracture. In general, the lattice specimens present a shear deformation failure at an angle of approximately 45° (Figure 3). This failure mechanism is in accordance with other results from the literature [9,27], and it could be predicted by using computational deformation. For each lattice specimen, the mean values of Young’s modulus, compressive strength, and strain are summarized in Table 3.

**Figure 4:**
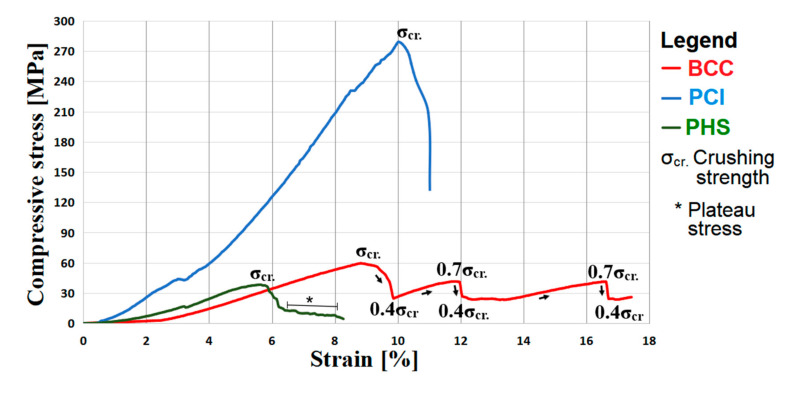
Stress–strain curves of lattice specimens.

Representative stress–strain curves are presented in Figure 4, where the crushing strength (σ_cr._) is marked. Three failure modes were recorded: brittle fracturing of the struts, successive cell collapse, and fracture propagation. Similar compressive failure modes were reported on lattice structures made of Ti6Al4V [19,25,27,28,37]. The first one appeared for PCI specimens, and it was characterized by a rapid fracturing of struts when the σ_cr._ was reached (Figure 4, colored blue). This brittle fracturing is also exposed in Figure 3a. PCI specimens display a brittle behavior because they have a higher diameter of struts and large voids.

The second mode of failure was recorded for BCC specimens. A typical stress–strain curve is shown in Figure 4 (colored red), where the strength was repeatedly lost and recovered as each layer collapsed and compressed into the one below. Practically, after reaching the σ_cr._, the BCC specimens lost 40% of the initial strength, followed by relatively uniform strengthening through densification up to 70% of σ_cr._ (see Figure 4, marked arrows). This cycle of lost and recovered strength was recorded just below 50% strain, but the stress–strain curve from Figure 4 was limited to simplify the chart. Even if the material of the lattice specimens has a brittle fracture with low plastic deformation (Table 3—fully dense parts), the BCC specimens have a lower stiffness due to a reduced diameter of struts. These specimens have a high number of lattice units (default of struts), contributing to a successive cell collapse and, thus, to a long fracturing process up to final failure.

The third type of failure was observed on PHS specimens (Figure 4, colored green). After the σ_cr._ was reached, plastic deformation starts as cells begin to yield, followed by continuous deformation at an approximately constant plateau stress. The plateau stress is marked in Figure 4, and some PHS specimens were fractured into many small pieces.

It is obvious from Figure 4 that the compressive failure mode of these lattice specimens is related to the strut size, cell design, and unit’s connectivity. The BCC and PCI topologies are isotropic designs, and the PHS design is an anisotropic one. This fact generates a significant variance of elastic behavior and compressive strength. Besides the strut’s diameter, an important variance is the number of nodes and the distance between them. For example, the BCC and PCI topologies were developed in such a manner that each node is connected to four other nodes, and the angle between every 2 struts is 90°. The distance between two nodes is approximately 0.4 mm for BCC and 1.4 mm for PCI structures. Due to the PHS design, the number of nodes from the specimen was significantly reduced, and the distance between them is greater (~4.2 mm).

As seen in Figure 4, the maximum strains of specimens derived from the load versus displacement curves revealed values between 5.9% and 10.2%. Compared with fully dense specimens, the BCC and PHS specimens have a significantly lower elastic modulus, ranging from 6 to 8 GPa. The PCI specimens are stiffer and have a Young modulus of about 28 GPa. The compressive strength varied from 38 to 279 MPa. These values are lower than those of solid specimens but are accompanied by a higher strain. As it was also demonstrated theoretically by FEA simulations (see Section 2.2), the PCI structures could archive a superior compressive strength to that of the PHS specimens.

From a medical point of view, the Young modulus of fully dense Ti6Al7Nb implants is three times higher than bone (see Table 3), and it could generate stress-shielding effect. In vivo studies demonstrate an accelerated osteointegration of Ti implants if their Young modulus is like the host bone [55,56,57]. The stiffness of Ti implants directly influence the activation and development of osteoblast cells at the bone–implant interface, and a reduced stiffness can improve the osseointegration process [21,58]. Due to their reduced Young modulus (or stiffness), the present lattice structures offer an important means to assign these structures to specific bone tissue or function (maxillofacial or hip). For example, the PCI specimens could correspond to cortical bone grafts (Table 3). On the other hand, the BCC and the PHS topologies could be applied in trabecular bone grafts for human femoral neck area. Because the physical–mechanical properties of human bones depend on various factors like health, sex, and age [59,60], these lattice structures offer knowledge for developing Ti6Al7Nb implants with physical–mechanical characteristics adapted to patients’ needs.

In the literature, various lattice structures have been SLM-manufactured, and their mechanical properties have been investigated. Table 4 summarizes some reported efforts undertaken to obtain a porosity level ranging from 49% to 70%, using typical lattice structures. As we argued above, the published studies are focused mainly on Ti6Al4V. Depending on the porosity level, lattice topology, and laser parameters, the compressive strength and Young modulus of Ti6Al4V specimens can be varied from 35 to 409 MPa and between 2 and 26 GPa [9,11,12,13,14,15,18]. From the compressive strength perspective, the PCI specimens had a similar value as those reported for Ti6Al4V lattice structures [9,11,18,29] and higher than others [12,13,14,15,19].

Comparing the stiffness of the lattice structures, we see the present results suggest that the BCC and PHS specimens are more elastic than previous outcomes [11,20]. On the other hand, previous SLM studies reported a stiffness even lower [12,13,14,15]. From the manufacturing point of view, Li et al. prepared titanium macro-porous specimens by a diffusion bonding method and reported the following results: a compressive strength from 35 to 68 MPa and a Young’s modulus between 2 and 5.5 GPa [61]. The actual BCC and PHS specimens have similar mechanical properties.

Under compressive tests, two types of fracture mechanism are known from the literature: a layer-by-layer one and a shear deformation at an angle of 45° [9,62,63]. The present results showed that the specimens failed in a brittle way by successive collapse of unit cell layers along 45° crush bands, and the strain ranged from 6% to 10%. This limited strain was also observed previously [1,31], and it could be improved by applying a heat treatment into an Ar-atmosphere. Likewise, the present study clearly shows that the lattice topology is a dominant factor which determines the mechanical response of SLM porous grafts. Even if the lattice specimens have a similar porosity, they will have unique elasticity and compressive strength in concordance with their topology.

### 3.3. Correlation between Gibson–Ashby Mathematical Model and Compressive Properties

To establish the *m* exponent, the values of Young modulus, compressive strength, and density for “fully dense” specimens were considered. The fully dense specimens were fabricated with the same process parameters, as in the case of the lattice structures described above. Hence, the *m* exponent was established for each lattice topology based on mechanical results. By applying the same value of *m* in Equations (2) and (3), we see that both compressive strength and Young modulus can be estimated (Table 5). In this manner, the Gibson–Ashby mathematical model was adapted to different lattice structures manufactured from Ti6Al7Nb powder via the SLM process.

As explained in recent studies, the Gibson–Ashby mathematical model for open cell foams indicates a linear correlation between the elastic modulus and the square of porosity [62,64,65]. Depends on the lattice geometry, it was found that the *m* coefficient can range between 0.1 and 4.0 [26]. Here, the values of *m* coefficient are 0.28, 0.41, and 1.72. The origin of this variance appears because the strut size, cell design, and unit’s connectivity differ. The mathematical correlation between elastic modulus–porosity (Equation (2)), respectively, between compressive strength–porosity (Equation (3)) is similar. For this reason, the *m* exponent has the same value in these equations, and it can be applied to estimate the theoretical compressive strength and Young modulus of each lattice topology. This fact proves that the elasticity and compressive strength of the lattice structures are dependent by the porosity level, in the same proportion. To predict the Young modulus and compressive strength of SLM implants made of Ti6Al7Nb with lattice structures integrated, the Gibson–Ashby mathematical model was adapted to each lattice type. This knowledge is useful for design analyses or optimizations.

### 3.4. SEM Analysis of Struts

**Figure 5:**
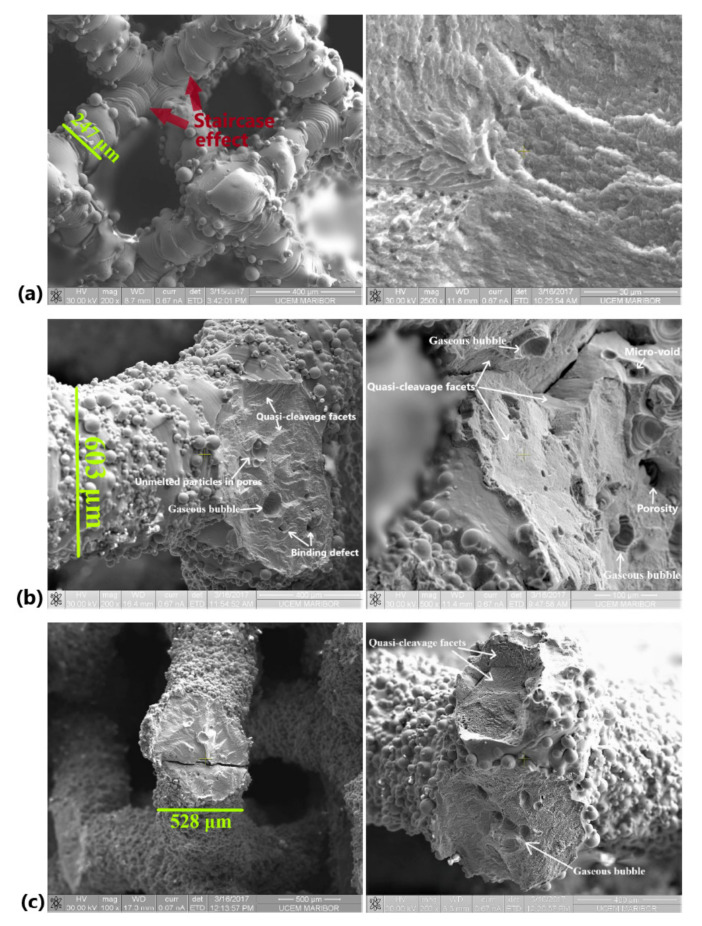
SEM analysis of strut fracture obtained after compression test: (**a**) BCC, (**b**) PCI, and (**c**) PHS. The white arrows indicate some fracture characteristics.

SEM analyses can provide details about issues or defects occurring on SLM specimens. The main manufacturing problems identified on strut surfaces were the stair-stepping effect, unmolten or half-attached particles on struts, and the balling phenomenon. Because the stair-stepping effect is dependent upon the build angle, it was clearly visible in BCC specimens. An example is presented in Figure 5a, marked with brown arrows. The BCC struts have a build angle of 45°, a value which is the minimum recommended to avoid the need for support structures. Moreover, this build angle significantly increased the staircase-shape profile. Due to their design of lattice units, the PCI and PHS specimens have a limited stairs profile (Figure 5b,c). Fine rippled structures exist on strut surface, as shown in Figure 5a.

It can be seen from the SEM images that all the Ti6Al7Nb struts were covered by a large amount of unmolten or partially melted grains. These particles are bonded on the strut’s surfaces, being nearly spherical, with a diameter of 20 to 80 µm (Figure 5). Two main causes can explain this behavior: the powder’s proximity to the melted area and the balling phenomenon. As reported in the literature [20,29], due to the proximity of powder to the melted area, there are inevitably insufficient melting grains which are incorporated into the strut surfaces. On the other hand, some of the particles which are near to the strut surface are caused by the balling phenomenon. This unfavorable phenomenon results in breaking up the liquid scan track during the laser irradiation, and it produces particles in spherical shapes [46,66]. Due to these two causes, the SLM struts are micro-rough; this finding has also been confirmed in the literature [8,10,25,37]. This micro-rough surface may decrease the mechanical properties. From the medical perspective, previous in vitro research observed that this typical micro-rough surface of SLM struts can promote cell attachment and proliferation by spreading pseudopodia [17,29].

Representative fractographs obtained on Ti6Al7Nb specimens are detailed in Figure 5 (right side images). The fracture morphologies confirm the brittle behavior of Ti6Al7Nb struts. In general, the cracks are close to the nodes network (Figure 5b,c). During rupture of the struts, shallow dimples are formed on quasi-cleavage facets, as shown in the larger magnification image from Figure 5a (right image). The main characteristics of fracture surfaces include smooth texture, quasi-cleavage facets, and closed pores, as depicted in Figure 5. The pores identified in strut fracture are micro-voids, gaseous bubbles, and binding defects. Inside of these pores, unmelted particles were also observed (Figure 5b). Together with metallographic analysis, all of these types of pores are described in the next section.

### 3.5. Microstructure

**Figure 6:**
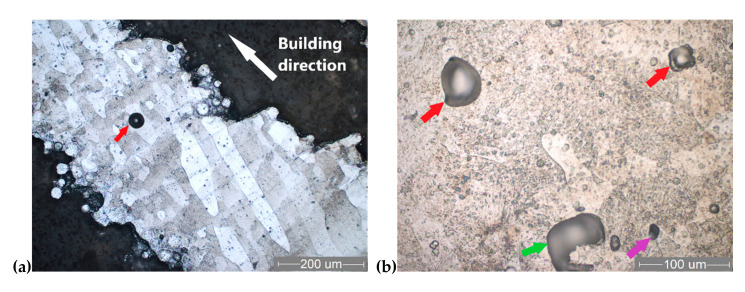
Optical micrographs of PHS specimens: (**a**) longitudinal section; (**b**) transverse section; and colored arrows indicate construction pores (Kroll’s reagent).

**Figure 7:**
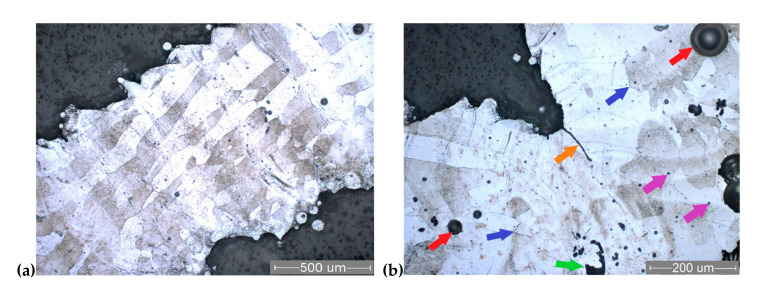
Optical micrographs of PCI specimens, longitudinal sections: (**a**) columnar grains orientation; (**b**) crack propagation after compressive test marked with orange arrow; colored arrows indicate construction pores (Kroll’s reagent)—micro-void (purple arrow), micro-crack (blue arrow), gaseous bubble (red arrow), and binding defect (green arrow).

**Figure 8:**
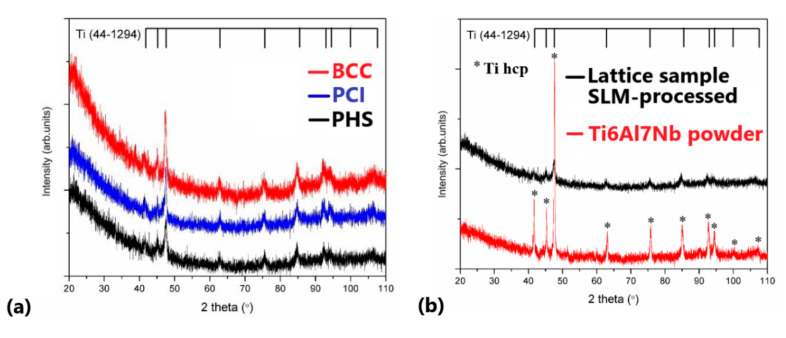
(**a**) Diffraction patterns of lattice specimens made of Ti6Al7Nb. (**b**) Comparison of diffraction patterns between raw powder and SLM lattice specimen.

Figure 6 and Figure 7 present typical metallographic images of the Ti6Al7Nb SLM-processed lattice specimens (PHS and PCI specimens). The structure consists mainly of solid solution-like grains, with a marked columnar shape that is specific to melting–crystallization processes such as SLM. The orientation of columnar grains is parallel to the build direction, and they are continuous through manufacturing layers (Figure 6a and Figure 7a). The length of columnar grains ranges from 100 µm to 1 mm (approximately 2–20 manufacturing layers). Etching of specimens has proven a chemical segregation both between grains and between the layers, making them obvious. Nevertheless, the crystal lattice is mainly of α type, as shown by XRD patterns (Figure 8). The columnar grains are perpendicular to the building layers they are crossing. This behavior was possible because each laser scanning leads to a partial remelting of the previously solidified layer directly underneath material and to the heating of its supporting layers to temperatures above the β-transus [35]. This effect is consistent with the well-known lamellar shape of β phase in titanium alloys. The crystalline microstructure of SLM specimens consists mainly of α’, the athermic martensitic form of α solid solution, indicating that the material was rapidly cooled from a temperature above the β transition. A similar microstructure consisting of acicular α’ martensite rather than equilibrium α and β phases was reported for the SLM specimens made of Ti6Al4V alloy [43,62,66,67]. At higher magnification (Figure 6b), a finely dispersed phase could be distinguished in the martensitic matrix. The finely dispersed phase could be a non-equilibrium intermetallic β-AlNbTi2 phase, inhomogeneously distributed along the build direction [39]. The occurrence of this phase could not be proven by XRD, as it appears in a less than 5% amount.

Due to staircase effect and particles stuck on struts, their micro-rough surfaces can also be observed in Figure 6a and Figure 7a. Typical SLM defects were recorded during the metallographic and SEM fractography analyses. These solidification defects appeared in all the struts, being classified as follows: micro-voids, micro-cracks, gaseous bubbles, and binding defects. Regarding the microstructural defects, some gas voids with 10–30 µm dimension can be observed in Figure 5b, Figure 6b, and Figure 7b (purple arrows). These spherical micro-voids are induced by gas entrapment [46]. The recorded micro-cracks are irregularly distributed in the strut structure, having different lengths (Figure 7b, blue arrows).

Large spherical pores can be seen, mainly in the center of the strut structure or lattice nodes. These gaseous bubbles are caused by the solidification shrinkage effect of the Ti alloy. Some gaseous bubbles are marked in Figure 6 and Figure 7 (red arrows). During the process, these were gas-filled, and their size ranged from about 50 to 140 µm.

The binding defects are irregular cavities associated with spherical particles, marked with green arrows in Figure 6b, and Figure 7b. As Liverani et al. explained, their formation mechanism could be related to the balling effect [68]. After the balling appeared, the new powder layer could not completely fill the irregular pores generated, and the laser energy may not be enough to melt the thicker powder layer. Besides the pores, a binding defect contained unmolten Ti6Al7Nb particles (see Figure 5b). Future studies are required to minimize or to avoid all of these manufacturing defects by optimizing the SLM process parameters for Ti6Al7Nb lattice structures.

A representative micrograph of crack propagation is illustrated in Figure 7b, where the rupture starts close to a node after the compression test. The crack propagation is marked with orange arrows in a longitudinal section. These are locations with maximum stress, and the same failure location was found in mechanical tests of Ti6Al4V scaffolds [1,63].

Figure 8a compares the XRD patterns of SLM manufactured specimens made of Ti6Al7Nb powder. The diffraction peaks correspond to hexagonal close-packed (hcp) titanium phase, and they are similar with the raw powder pattern (Figure 8b). The SLM process and design do not cause changes in the material crystalline structure or induce any secondary peaks of rutile or anatase phases. Thus, the mechanical properties of the lattice specimens were not affected by significant oxidation during the fabrication. This is a positive result, since the objective of this work was to test the mechanical properties of different lattice specimens manufactured by the same pulsed-laser melting parameters. The hcp pattern can be attributed both to the α solid solution and to the α’ martensite, similar as found by Chlebus and Attar [35,66]. On the other hand, it was observed that the maximum intensity diffraction is better presented for the BCC specimen because its structure is more compact (Figure 8a). Therefore, if the voids in the lattice are more important, the quality of the signal recorded on the diffractometer will decrease accordingly (e.g., compact powder vs. BCC specimen, Figure 8b).

## 4. Future Perspectives

Based on these findings, the following future studies are approachable. From a cell-design point of view, future research should use a mathematical algorithm to generate and optimize the PCI and PHS topologies, and to define the relationship between the relative density and the cell aspect ratio. The established process parameters led to a good and stable manufacturing, but to limit the solidification defects from Ti6Al7Nb struts, extensive investigations should be developed to optimize the laser parameters. Nevertheless, additional work is required to evaluate the fatigue resistance of these lattice structures made of Ti6Al7Nb.

On the other hand, the obtained porosity, the reduced Young modulus of these lattice structures, and the micro-rough surface of struts could improve the osseointegration process. These three aspects could allow a fast and effective bone ingrowth, aiming toward the long-term stability of implants. Future in vitro and in vivo study is needed to evaluate the cells’ behavior and bone ingrowth, using the present lattice structures SLM-manufactured. This comparative study focused on three topologies of lattice structures made of Ti6Al7Nb, offering preliminary knowledge which could enhance the development of medical treatments in different sectors, such as orthopedic, craniomaxillofacial reconstructions, and regenerative medicine. The results could contribute to expand the concept of implant customization from a structural and mechanical point of view, according to patient’s age and health status.

**Figure 9:**
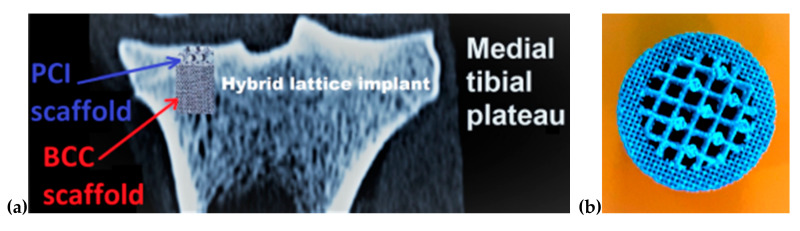
Concept of hybrid lattice implant: (**a**) Simulation done on computed tomography (CT) image of proximal tibia (coronal section); (**b**) SLM-manufactured from Ti6Al7Nb powder (border—BCC scaffold, and middle PCI—scaffold).

The knowledge exposed could be used to develop hybrid lattice implants. This concept means that the medical component will have distinct physical–mechanical properties for the cortical bone and trabecular tissue, avoiding the effect of stress shielding or overburdening. A practical example of this future perspective is presented in Figure 9, where a hybrid lattice implant is illustrated. It contains a BCC structure for trabecular host tissue, respectively, and a PCI structure in cortical region. To accelerate the osseointegration process, these hybrid lattice implants offer the possibility of infiltrating or coating them preoperatively with bioactive materials such as hydroxyapatite, bisphosphonates, simvastatin, or antibiotics [69,70,71,72,73]. Moreover, these lattice structures can be seeded preoperatively with stem cells obtained from patient adipose tissue. Under laboratory conditions, the stem cells could multiply on the metallic lattice structure, forming an ideal implant without any risk of rejection. All these medical possibilities should be analyzed in future studies.

## 5. Conclusions

The established SLM parameters allowed for stable manufacturing, and the Ti6Al7Nb specimens were fabricated with 56% porosity. When we compare them with their fully solid counterparts, we see that the present lattice specimens reduced the processing time and powder consumption. Under compression tests, three failure modes were recorded. Depending on lattice topology, the compressive strength ranged from 38 to 279 MPa, and the elastic modulus was between 6 and 28 GPa, being 73–94% lower than for solid specimens. Even if the porosity level of lattice structures is similar, their compressive strength and Young modulus are influenced by the cell design and strut diameter. The results of this work proved that it is possible to SLM manufacture lattice structures made of Ti6Al7Nb with comparable physical–mechanical properties to Ti6Al4V specimens. To predict the Young modulus and compressive strength of Ti6Al7Nb specimens, the Gibson–Ashby mathematical model was adapted to each lattice topology.

The dimensional deviations of struts are ±0.05 mm, and their surfaces are covered by a large amount of partial melted grains. Some solidification defects were recorded in struts’ microstructures. Because the specimens were tested in as-build condition, the fractographs revealed a brittle rupture of struts near to nodes and a crack orientation at 45°. The microstructure has shown mainly α’ martensite, with columnar grains. The diffraction peaks correspond to hcp titanium phase, and they are similar with raw powder pattern. To exploit the potential of these lattice structures made of Ti6Al7Nb, future work should investigate the osteoblast cell affinity, in vivo osseointegration feasibility, and design optimization.

## Figures and Tables

**Table 1:** Selective laser melting (SLM) parameters configured to manufacture the Ti6Al7Nb lattice specimens.

Laser Power (W)	Scanning Speed (mm/s)	Hatch Distance (mm)	Spot Size (µm)	Layer Thickness (µm)	Energy Density (J/mm^3^)
180	300	0.11	45	50	109.1

**Table 2:** Dimensional assessment of designed strut diameter and actual (mean ± standard deviation).

Lattice Topology	Nominal Strut Diameter (mm)	Actual Strut Diameter (mm)
BCC	0.25	0.29 (±0.04)
PCI	0.50	0.55 (±0.05)
PHS	0.45	0.49 (±0.04)

**Table 3:** Physical–mechanical properties of Ti6Al7Nb lattice specimens SLM-manufactured (mean ± standard deviation).

Physical–Mechanical Characteristic	Fully Dense	BCC	PCI	PHS	Cortical Bone *	Trabecular Bone *
Young Modulus (GPa)	104 (±3.8)	8.2 (±1.7)	28.6 (±3.6)	6.1 (±1.2)	10–29	0.8–5
Compressive Strength (MPa)	790 (±26)	59.4 (±6.4)	279.0 (±27.5)	38.8 (±4.7)	135–205	2–7
Compressive Strain (%)	1.5 (±0.2)	8.9 (±1.6)	10.2 (±2.6)	5.9 (±1.1)	1–5	up to 1

* Properties of human femur or tibia bone [51,52,53,54].

**Table 4:** Comparison regarding the obtained physical–mechanical properties of lattice specimens SLM-manufactured.

Material	Lattice Topology	Porosity (%)	Strut Diameter (mm)	Compressive Strength (MPa)	Strain (%)	Young Modulus (GPa)	Source
Ti6Al7Nb	BCC, PCI, PHS	56	0.3–0.6	38–279	6–10	6–28	This study
Ti6Al4V	Cubic, Twisted	49–66	0.9–1.8	215–409	2.8–4.2	10–26	[9]
	Simple cubic	63.8	0.5–0.6	219	N/A	8.7	[11]
	Schwartz primitive, Cylinder grid	70	0.3–0.6	120–140	10–12	1.9–2.4	[12]
	Diamond	65	0.3–0.4	99–150	10	3–4	[13]
	Reinforced BCC and FCC	65	0.3	35–140	8–13	2–3	[14]
	Dodecahedron	65–69	0.20–0.25	78–117	4–5	2.6–3.5	[15]
	Diamond	55–65	0.6–1	68–228	N/A	4.2–8.3	[18]

N/A indicates that the value is not available.

**Table 5:** Values of *m* exponent applied to predict the Young modulus and compressive strength of each lattice structures designed and SLM-manufactured from Ti6Al7Nb.

Physical–Mechanical Characteristics		BCC	PCI	PHS
**Actual Porosity (%)**		~56		
**Pore Size (mm)**		0.40–0.51	0.85–0.91	0.50–0.62
**Value of *m* exponent**		0.41	1.72	0.28
**Young modulus (GPa)**	Theo. (Equation (2))	8.1	33.9	5.5
	Exp.	8.2	28.6	6.1
**Compressive strength (MPa)**	Theo. (Equation (3))	61.5	258.2	42.0
	Exp.	59.4	279.0	38.8
